# A Series of Cases, Exhibiting the Effects of Pressure in Cancerous and Other Diseases

**Published:** 1821-01

**Authors:** Samuel Young

**Affiliations:** Surgeon to the Cancer Institution, Gerard-Street, Soho; Member of the Royal College of Surgeons, and of the London Medical and Chirurgical Society, &c.


*A Series of Cases, exhibiting the Effects of Pressure in Cancerous and other Diseases.* By SAMUEL YOUNG, ESQ. Surgeon to the Cancer Institution, Gerard-street, Soho; Member of the Royal College of Surgeons, and of the London Medical and Chirurgical Society, &c.

[Continued from vol. xliv. page 364.]

THE circumstance of the existence of fungus under a diseased state of integument in cancerous and other morbid alterations and growth, being frequently mistaken for the presence of matter or pus, was generally alluded to in the last article of this series, when the cases with which we have been favoured by Mr. Trowbridge were reported; and since, in practical application in many instances, much mischief may be produced, or avoided, according to the means employed, a further consideration of the subject in this place may not be unimportant; which, on the contrary, would have been too digressive in the midst of the report of the cases just alluded to.

This state of fungous disease is frequently very deceptive, and, to usual observation and tests, leads to the conclusion that matter has been formed; and, in some cases, the life of the patient will inevitably be lost, if an incision be made for the purpose of evacuating the supposed fluid; while, in all of such instances where an opening is made, mischief in various degrees must necessarily result, without a possibility of any attendant good.

We are very apt to follow a common routine practice without reflection, and consequently without principle; and we probe, and cut, and poultice, mechanically. As a necessary preliminary, a formality not to be dispensed with, a surgeon frequently probes a wound at first sight, for no known purpose of good, and quite innocent of any rational prospect of it; though really guilty of giving unnecessary pain, and running the risk of much probable mischief, to his patient. Such unnecessary interferences and meddlings were called, by my good friend the late Dr. Denman, most justly and appositely, "the impertinencies of surgery."

In following this sort of dull, unmeaning, and unprincipled routine work of practice, if I know several cases, during part of my life, where I have done mischief, I think it is pretty clear I must have done a great deal more of which I have been wholly ignorant; and, as I feel assured, at least in this instance, that I do not stand *alone* with the profession, my reflection and repentance on this subject may prove useful to others.

Poultice, experience sufficiently shows, is only the nurse and feeder of disease in many instances: even in common abscess, its undue employment is a great abuse, and leads to much mischief. And yet how indiscriminately it is used,—even to court suppuration, which might, and (generally speaking, if possible,) ought, always to be avoided; and even in instances where there is an evident growth of morbid structure,—the increase of which it must necessarily favour,—still we see the poultice, in conjunction with fomentations, a common practice.

And then, in heedless uniformity with the rest, and in completion, a lancet or knife is plunged into any part that may afford the appearance of a point, as in common abscess; and thus a door is opened for new, and perhaps irremediable, mischiefs; and the protrusion and increased growth of a fungus, instead of the escape of pus, is the consequence.

In all equivocal cases the great object of good practice is to preserve, if possible, the integument entire, and reduce the action of the vessels of the part. The common routine of fomentations and poultices will, generally speaking, only accelerate disease; in some instances, produce fatal mischiefs, which might have been avoided; and, in all, augment, at least, the evils of the case. I speak most decidedly from facts and my own experience. A lady, for example, about eight years ago, had a small and most troublesome tumor at the angle of the jaw under the right ear. The whole of the covering integument became discoloured and diseased, was drilled through like a cullender with small holes, most exquisitely sensible to the touch, affording but very spare suppuration, generally of a very ichorous and irritating nature; and the entire character of the disease was of a very malignant, though equivocal, nature, and taking on, in the earlier stages, the appearance of abscess. It was however, after a most obstinate resistance, entirely reduced and removed under the treatment of pressure.

Some time after, the lady being then in the family-way, a small tumor appeared upon, and just about the centre of, the breast-bone. It increased, though not very rapidly, to a considerable bulk; became discoloured at its apex, where it afforded the appearance of a point as in common abscess, as well, ultimately, as the evidence to the feel of the existence of pus. It was poulticed and fomented according to the usual routine of practice; and, finally, an opening was made to discharge the contained matter. This was done in conjunction with a very intelligent practitioner, who has great celebrity as a physician and surgeon in the country where he lives. In making the opening, I found very considerable resistance, not of the integument, but when the abscess-lancet had penetrated, and cutting downwards and upwards to enlarge the opening, as if tough cords or bands were opposed internally to the edge of the instrument. Nothing but a thickish, and rather dark-coloured, blood followed, somewhat copiously, this attempt. My professional friend, just mentioned, then took the lancet from me, and made a deeper incision, thinking I had not sufficiently penetrated into the cavity of the supposed abscess; but nothing followed this attempt but a further discharge of the same sort of blood as issued on the first incision, except here and there a flake of whitish matter, denoting something of a curdy or scrofulous nature. Poulticing, in almost all its varieties, was tried in this case to no effect, except a bad one: the growth of an immense fungus was the result, and which ultimately destroyed the patient after long and great suffering.

During the pregnancy of this lady, the progress of the disease was comparatively slow, and evidently suspended. But, after her delivery, (the child, and especially in contrast to the then state of the mother, being remarkably fine and healthy,) the growth and ravage of the disease became so striking and formidable as to exclude every hope of relief.

It is rather singular (not indeed rather, but, according to my present view of things, strangely and most infatuatedly stupid,) that the treatment by pressure of the breast-disease in this patient never once occurred to me, although I had but shortly before used it, and with success too, to the disease at the angle of the jaw;—diseases similar in both instances, if not precisely alike in their natures. But at that time I was entangled in the common practice, and, although I had applied it with success in a few instances of breast-diseases, the treatment by pressure had not been contemplated by me, even in theory, by any means to the extent of its application that it has been since; and which, in practical application, the experience and absolute knowledge of its beneficial results, has since been almost daily enlarging and confirming.

Such and similar instances as this case affords, have made an impression on my mind, and resolved it to contemplate and consider things well before a plan of treatment is adopted. Not to do mischief, is no small perfection in our profession, as daily experience shows; and it is incomparably better to do nothing than to do harm.

How far the mischiefs of the chest-sore, in the case just stated, might have been wholly avoided by a different and very opposite course of treatment being adopted, may, in this individual instance, be equivocal. But there can be neither doubt nor question but that the whole of them were accelerated and aggravated by the plan that was adopted; and that, if the integument had been supported by compress, and bandage in conjunction with the evaporating treatment, the disease would have been retarded and modified, at least, if not entirely suppressed and removed. And, further, neither question nor doubt can be entertained, that to have done nothing would have been incomparably better in this case than doing that which was done.

In other cases, however, a plan of poulticing, &c. must inevitably prove fatal; and which might be entirely avoided, as experience shows, by adopting the opposite plan of compression, &c. In the case, for example, of the late Duke of Buccleugh's female domestic, the last reported case in the *Further Minutes, &c.* published in 1818, and so frequently referred to, had the disease proceeded but a few weeks longer, a discoloured point at the top of the breast would have presented all the common tests of the existence of pus; and if, contrary to the history of the case and all good practice, an opening had been made, as we see in common practice most probably it would have been, for the purpose of discharging the (supposed) contained fluid, the patient would have been inevitably lost by the aggravation and ravage of the fungoid disease, which then existed.

I have frequently had to observe, on various occasions, as far as my own observations have extended, in cases where any thing of an equivocal and mixed nature has been shown, as well as in the more decided instances of schirri, the evil results from leeching, fomenting, and poulticing; and up to the present period, on a large ground of experience, the accumulated mischiefs so decidedly resulting and originating from such practice, authorize from me the most unqualified reprobation of it.

Leeches by their bites disease the skin, with, in no instance, any permanent relief, and scarcely with any temporary alleviation, while they hurry on to the formation of tubercular disease; the first to form it, but the very last to yield. Fomentations and poultices, as already observed, only tend to weaken the integument, already too weak to resist disease; and, by giving laxity and facility of circulation, only accelerate the growth of that which ought to be the object of all rational practice to suppress. Take, for example, the first case as reported by Mr. Trowbridge, with the many score of similar cases I have seen, and ask what would have been the result of leeching, fomenting, poulticing, &c.?—The inevitable aggravation of a destructive and fatal disease.

In making this summing-up, I give it with all due respect to practical truth, after the most impartial and correct observation my mind is capable of, authorized by a large, but not a boasted experience; and, if *positively* given, that *positiveness*, let it be understood, is the result of conviction, not presumption,—of errors self-acknowledged and corrected: and, as I feel my errors have been most brotherly in common with the profession at large, I have only to say, let there be a general confession, and let us amend our lives.

I shall now take leave to state a case from the many of a mixed sort,—*i. e.* where common inflammatory symptoms of the part are conjoined with the more specific or morbid progress of cancerous alteration, and where the beneficial results of a judicious practice by pressure were decided and conspicuous.*

Before, however, the detail of this case is given, it will be proper to remark, that the very formidable case just alluded to, of the late Duke of Buccleugh's female servant, has gone through the test of several years in the confirmation of perfect recovery. Since the report of her case, she has married, become a mother, and suckled the child freely with the formerly diseased breast. This information has been the result of frequent communication from the party. Shortly after her delivery, the sister of the patient called upon me, stating that a swelling or hardness had come on, on the formerly diseased breast; of which (although her medical attendant attributed it to the common occurrences in such cases,) they were anxious I should be informed. I merely ordered the acetated ammonia and spirituous lotion to be used, with a request that, if the swelling or hardness did not subside, or any difficulty of suckling should be experienced, that I should be immediately informed of the circumstance. I heard no more of the case then. A twelvemonth, as nearly as I can recollect, has now elapsed; and I have since heard that the patient has been perfectly well, and other patients have been recommended from the same source.

[*To be continued.*]

* This case will be inserted in the next Number.—EDIT.

